# Multiscale Analysis of Impact-Resistance in Self-Healing Poly(ethylene-co-methacrylic acid) (EMAA) Plain Woven Composites

**DOI:** 10.3390/polym16192740

**Published:** 2024-09-27

**Authors:** Zhenzhen Zhang, Ying Tie, Congjie Fan, Zhihao Yin, Cheng Li

**Affiliations:** School of Mechanical and Power Engineering, Zhengzhou University, Science Road 100, Zhengzhou 450001, China

**Keywords:** EMAA, self-healing, multiscale, low-velocity impact (LVI)

## Abstract

A study combining multiscale numerical simulation and low-velocity impact (LVI) experiments was performed to explore the comprehensive effects on the impact-resistance of EMAA filaments incorporated as thermoplastic healing agents into a plain woven composite. A multiscale micro–meso–macro modeling framework was established, sequentially propagating mechanical performance parameters among micro–meso–macro models. The equivalent mechanical parameters of the carbon fiber bundles were predicted based on the microscopic model. The mesoscopic representative volume element (RVE) model was crafted by extracting the actual architecture of the monolayer EMAA filaments encompassing the plain woven composite. Subsequently, the fiber and matrix of the mesoscopic model were transformed into a monolayer-equivalent cross-panel model containing monolayers aligned at 0° and 90° by local homogenization, which was extended into a macroscopic equivalent model to study the impact-resistance behavior. The predicted force–time curves, energy–time curves, and damage profile align closely with experimental measurements, confirming the reliability of the proposed multiscale modeling approach. The multiscale analysis reveals that the EMAA stitching network can effectively improve the impact-resistance of plain woven composite laminates. Furthermore, there exist positive correlations between EMAA content and both impact-resistance and self-healing efficiency, achieving a self-healing efficiency of up to 98.28%.

## 1. Introduction

In contrast to metallic materials, carbon fiber-reinforced polymer (CFRP) composites find widespread applications in aerospace as well as transportation fields due to their high specific stiffness, specific strength, and commendable mechanical properties [1,2,3,4,5]. CFRP composites can effectively reduce the weight of aircraft and vehicles, which improves dynamic performance and fuel efficiency. However, damage is easily induced by impacts from external objects during service life, which greatly reduces the stiffness and strength of the composites [6,7,8,9,10].

The high cost of composites greatly reduces the feasibility of total replacement, and traditional repair methods are not effective at repairing internal damage. Composites with self-healing functionality can recover their load transfer capacity after damage [11,12,13]. This healing can take place autonomously after the damage has occurred or it can be activated with the help of external conditions (heating, pressurization, radiation, etc.) [14]. Therefore, engineering materials with self-healing functionality are of great value in practical applications. On the one hand, self-healing can lengthen the useful life of materials and lower maintenance expenses; on the other hand, it can encourage the use of resources and materials in a sustainable way.

The thermoplastic material poly(ethylene-co-methacrylic acid) (EMAA) has been shown to be an excellent healing material, exhibiting good healing properties [15,16,17]. Yang et al. [18] stitched filaments of EMAA into a composite laminate, giving the composite laminate high resistance to delamination and self-healing. In this structure, EMAA was delivered to the damaged area through a 3D stitching network, realizing self-healing functionality in the composite laminate. Through a double cantilever beam (DCB) test, it was found that the EMAA stitched laminate could realize self-healing with up to 150% fracture toughness. Pingkarawat et al. [19] investigated the influence of stitch density on both delamination toughness and the self-healing capabilities of materials. The results showed that an increase in EMAA stitch density increases the inter-laminar fracture toughness accordingly. Varley et al. [20] added EMAA in the form of a nonwoven fabric into the middle of a multilayer carbon fiber prepreg to investigate the healing mechanism and the effectiveness of its recovery performance after Mode I and Mode II damage, and the results showed that recovery performance after Mode I could be more than 200%. Caballero et al. [21] used a novel EMAA implantation method to spray EMAA nanoparticle dispersions on the surface of a specimen. It was shown that the EMAA particles were able to be uniformly distributed on the fiber surface and exhibited good adhesion, and the coating also resulted in a 260% increase in inter-laminar fracture toughness. These studies show that the addition of EMAA has a significant healing effect on composites, resulting in laminates with high delamination resistance and self-healing ability. However, most past studies have focused on unidirectional composites and DCB tests, and scant consideration has been given to the effect of the addition of EMAA stitching on the impact-resistance of plain woven composites as well as on the healing effect after impact. Hence, further research on the impact-resistance of EMAA stitching within plain weave composite materials and exploring its healing effect is of great significance.

Moreover, the realm of self-healing plain weave composite laminates remains relatively unexplored in simulation studies, thereby warranting a necessity for investigations in this domain. Multiscale analysis is commonly used to investigate multilevel behavior in materials [22,23,24,25,26,27,28]. Li et al. [23] integrated macroscopic and microscopic scale simulations using a multiscale analysis approach, facilitating interactions by exchanging information regarding intrinsic behaviors. In a similar vein, Yang et al. [25] applied the finite element method for micro-scale fiber yarn modeling and mesoscale fabric representation. Employing two-scale RVEs, they forecasted the overall damage response of 8HS woven composites when subjected to tensile loading. Zhang et al. [26] proposed a multiscale model utilizing a global–local modeling approach to prognosticate progressive damage and damage response in hybrid 3D textile composites under conditions of three-point bending. Under the mesoscale model, the matrix and fibers in the polymer are represented by repeat unit cell (RUC). These investigations underscore the efficacy of the multiscale modeling methodology in evaluating composite mechanical attributes.

This study systematically conducts low-velocity impact (LVI) tests on plain woven composite laminate panels by introducing EMAA stitches along the laminate thickness direction. The impact-resistance of EMAA self-healing plain woven composite laminates (EWL) is investigated at different impact energies and stitching spacing, both before and after self-healing, to evaluate its self-healing efficiency. In numerical simulation, since the EWL space configuration is complex and a single macroscopic model ignores its inhomogeneity, a multiscale model of EWL is developed on three scales: micro–meso–macroscopic. At the microscopic scale, a microscopic model is constructed based on the structure of carbon fiber bundles. At the mesoscopic scale, the direct periodic arrangement of mesoscopic RVE to construct a macroscopic model for numerical simulation causes great computational costs. The macroscopic model is constructed with high accuracy and low computational cost through a local homogenization-based scheme. The accuracy of the multiscale model is validated through comparison with experimental findings.

## 2. Experimental Procedure

### 2.1. Specimen Preparation

The raw material of the EMAA filaments was Nucrel 960 pellets produced by USA DuPont Ltd. (Wilmington, DE, USA). Prior to use, the EMAA pellets were dried in an oven (DZF-630AB, Zhongxingweiye, Beijing, China) for 48 h and then heated to achieve a molten state at 180 °C. Through meticulous control of outlet diameter and haul-off speed during the EMAA extrusion process in a twin-screw extruder (SJZS-10B, Ruiming Experimental Instrument Manufacturing Co., Ltd., Nanjing, China), the EMAA filaments attained a diameter of 1.2 mm. This value was determined to achieve self-healing in the composites without significantly sacrificing in-plane performance. Plain woven composite prepregs were supplied by Weihai Guangwei Composite Material Co., Ltd. (Weihai, China). The prepregs were composed of carbon fibers T300/3K (density: 1.78 g/cm^3^, diameter: 7.0 μm) and epoxy resin 7901 (density: 1.20 g/cm^3^), and their relevant mechanical parameters are shown in Table 1.

**Figure 1:**
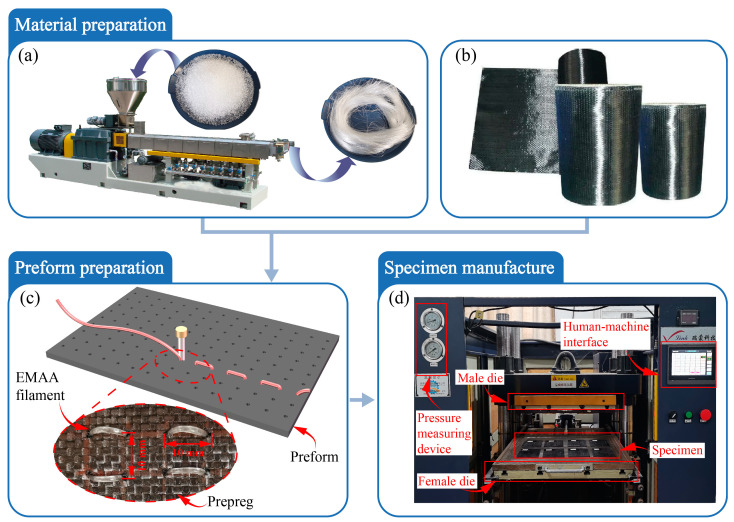
Illustration of specimen preparation: the material preparation including (**a**) EMAA filament and (**b**) the prepreg, as well as (**c**) the preform preparation; (**d**) the manufacturing process.

The prepregs were cut into rectangular shapes with a size of 150 × 100 mm, each group containing 12 layers, with a thickness of 0.25 mm per layer. Eight layers of intermediate layer prepregs were stitched along the thickness direction with EMAA filaments, and the planar stitches were spaced at 10 mm to create a three-dimensional self-healing channel. It is noted that a large stitch spacing might cause the EMAA healing agent to be too far from the damage cracks, thus significantly reducing the healing efficiency. Meanwhile, the composites could possess a severe degree of damage when the stitch spacing is too small, leading to a potential reduction in impact-resistance. After balancing these considerations, the stitch spacings were set to 10 mm, 15 mm and 20 mm in this study. Then, two layers of prepreg were applied to the top and bottom surfaces to prevent any loss of EMAA during the processes of high-temperature curing and heated self-healing. As shown in Figure 1, the specimens were put into a QMG-100 thermoforming machine and hot-pressed to manufacture EWL. Simultaneously, 12 layers of unstitched prepreg were thermoformed into non-EMAA stitched plain woven composite laminates (NWL).

### 2.2. Low-Velocity Impact Test

**Figure 2:**
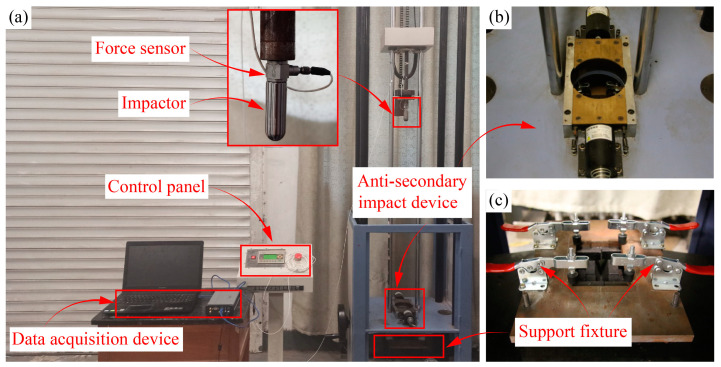
The testing platform for conducting the LVI tests. (**a**) the testing platform used to carry out the LVI tests, (**b**) the anti-secondary impact device and (**c**) the support fixture.

A LVI test platform was constructed in accordance with the ASTMD7136/D7136M-20 standard [29]. The testing platform consisted of several components, including an impactor, a guide rail, an anti-secondary impact device, a control panel, and a data acquisition device, as depicted in Figure 2.

At the beginning of the test, the required impact energy was achieved by releasing the impactor from a predetermined height directly above the positioned specimen. The impactor descended freely, making contact with the specimen’s center and imparting the desired impact energy. The impactor possessed a mass of 2.5 kg and a radius measuring 8 mm. The experimental testing regimen included impact energies of 10 J, 15 J and 20 J.

Initially, the impactor was dropped from a specified height to achieve the desired impact energy. During the LVI tests, an ICP208C03 dynamic force sensor recorded and collected the instantaneous impact force *F*(*t*). Subsequently, the displacement-time *ξ*(*t*) and impact energy-time *E*(*t*) values were plotted using the obtained data:

(1) $$\xi \left(t\right)=\int_{0}^{t}\left[v_{0}-\int_{0}^{t}\frac{F\left(t\right)}{m}dt\right]dt$$

(2) $${E(t)=E}_{k0}-E_{k}(t){=E}_{k0}-\frac{1}{2}m{\left[v_{0}-\int_{0}^{t}\frac{F\left(t\right)}{m}dt\right]}^{2}$$

where *v*_0_ and *m* refer to the initial impact velocity and the mass of the impactor, and *E_k_*_0_ and *E_k_*(*t*) are the initial and transient kinematic energies, respectively. As the input parameter, the impact energy *E_k_*_0_ is set to 10 J, 15 J and 20 J. It is noted that the energy dissipation due to friction was not considered. Thus, the values of initial impact velocity, *v*_0_, are ideally calculated as 2.828 m/s, 3.464 m/s and 4 m/s in the aforementioned impact energy cases. In this study, five replicates were included in each testing group to ensure the reproducibility of the measured data, and the median curve was selected to represent the LVI response of the specimen.

### 2.3. Self-Healing Test

Following the first impact, a protective release film was applied to the top and bottom surfaces of the EWL specimens. Subsequently, the EWL specimens were reinserted into the thermoforming machine and subjected to a temperature of 150 °C for a duration of 30 min. This was followed by low-pressure consolidation at ambient conditions (20 kPa) for a period of 10 min, promoting the activation of the self-healing mechanism. Throughout the process of self-healing, a reaction occurs between the activated amino and carboxyl groups within the EWL to generate water. Under the temperature of the thermoforming machine, the generated water transforms into high-pressure bubbles, forming a pressure conduction mechanism that pushes EMAA to flow into the cracks of the damaged area, thereby producing a healing effect on the damaged area.

## 3. Multiscale Modeling of EWL

### 3.1. Microscale RVE Modeling

**Figure 3:**
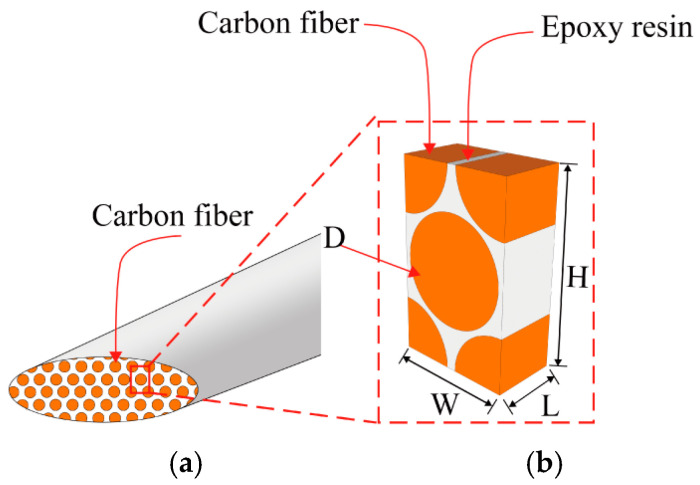
Schematic illustration of the construction of the microscopic model: (**a**) fiber bundle, (**b**) RVE model.

At the microscale, fiber bundles are typically regarded as unidirectional composites of carbon fiber/resin matrix exhibiting transverse isotropy. These fiber bundles are uniformly distributed within the matrix, forming a hexagonal pattern, with their cross-sections assumed to be circular [30]. Figure 3 shows the microscopic RVE geometric model, where the carbon fiber volume fraction *V_f_* is 0.8. The dimensions of the RVE model follow a ratio of length (*L*), width (*W*), and height (*H*) of L:W:H =1:1:$\sqrt{3}$, while the geometric parameter *D* represents the diameter of the carbon fiber, D:L =0.94:1.

In the microscopic RVE model, the maximum principal stress damage criterion was extensively utilized to define the onset of damage in both carbon fibers and the matrix. Furthermore, a damage evolution law based on fracture toughness was employed to govern the degradation of material properties across distinct failure modes. Consequently, the material strength and stiffness would progressively decline with escalating levels of damage.

The UMAT subroutine was written based on the above damage criteria. By adding periodic boundary conditions [31] to the microscopic RVE model, six loading modes were applied: tension and compression in the warp direction, tension and compression in the weft direction, in-plane shear, and out-of-plane shear. The microscopic RVE model equivalent stress–strain response was obtained, thus obtaining the equivalent mechanical properties of the microscale RVE model, as listed in Table 2.

### 3.2. Mesoscale RVE Modeling

**Figure 4:**
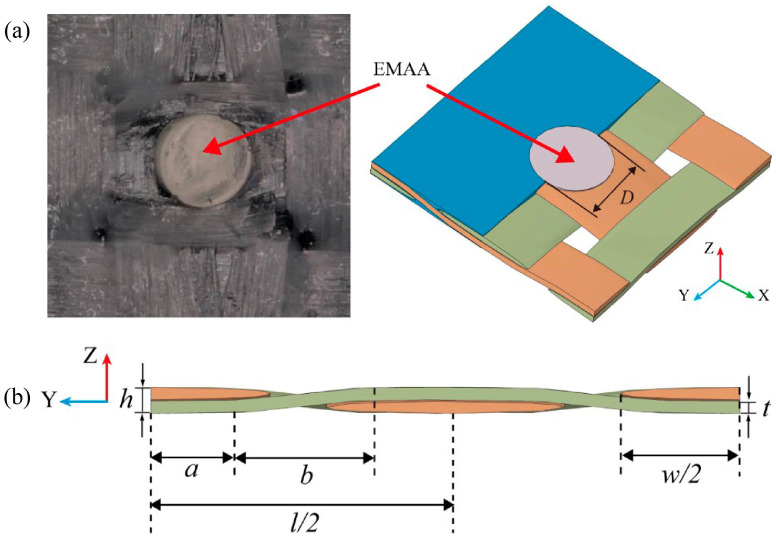
Schematic illustration of local high-fidelity mesoscopic RVE model structure: (**a**) top view, (**b**) lateral side view.

Typically, the overall structure of the composite was considered to consist of a periodic arrangement of mesoscopic RVEs. The presence of EMAA stitches introduces a discernible influence that interacts with both the fiber bundles and the matrix. By observing the EWL structure, extracting EMAA and the fiber bundles and matrix influenced by EMAA (stitch-influenced area), a local mesoscopic RVE model was constructed, as shown in Figure 4a, where the fibers in the X and Y directions represent the warp and weft fibers, respectively. In the center is the inserted EMAA filament with a diameter of 1.2 mm.

Plain-woven composites consist of a yarn and a matrix. Their mechanical properties are influenced not just by the mechanical properties of these individual components, but also by the specific geometry of the internal warp and weft cross-sections, as well as by the pattern of the weave. The woven paths of the fibers in the mesoscopic geometric model were simplified and equated to a combined path containing straight lines and sinusoidal curves. Additionally, the cross-sectional shape of the fibers was simplified and equated to an ellipse, the woven structure and parameters are shown in Figure 4 and Table 3. Therein, *l* and *h* are the length and height of the RVE, *w* and *t* are the width and height of the cross-section of the fiber bundle, and *D* is the diameter of the stitch. Moreover, *a* and *b* are the characteristic parameters defining the shape of the fiber bundle.

In the EWL local mesoscopic RVE model, EMAA employs the ZWT [32] nonlinear viscoelastic model:

(3) $${\sigma (t)=E}_{0}\varepsilon +\alpha {\varepsilon^{2}+\beta \varepsilon^{3}+E}_{1}\int_{0}^{t}\dot{\varepsilon }(\tau )\exp\left(-\frac{t-\tau }{\theta_{1}}\right)d\tau +E_{2}\int_{0}^{t}\dot{\varepsilon }(\tau )\exp\left(-\frac{t-\tau }{\theta_{2}}\right)d\tau$$

where *σ* and *ε* are the stress and strain, $\dot{\varepsilon }$ is the strain rate, *E*_1_, *E*_2_ and *θ*_1_, *θ*_2_ are the elastic constants and relaxation time, respectively.

Matrix damage initiation was determined through the maximum principal stress criterion, while carbon fiber damage initiation employed the three-dimensional Hashin failure criterion. The subsequent stiffness degradation accurately characterizes both damage initiation and the subsequent damage evolution process. EMAA participates solely in calculations without determining the state of damage. The above damage criteria were written as a UMAT user subroutine, and multiple displacement loads were applied to the local mesoscopic model through periodic boundary conditions. Six types of loading were included: warp tension, compression, weft tension, compression, in-plane shear, and out-of-plane shear. The mechanical properties of the matrix, along with those of the fiber bundles employed at the local mesoscopic scale, are listed in Table 1 and Table 2.

### 3.3. Local Homogenization Method

**Figure 5:**
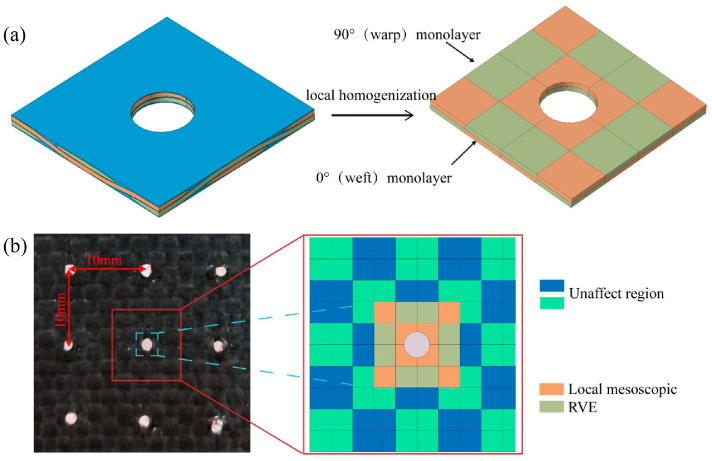
Illustration of: (**a**) transformation of local high fidelity mesoscopic RVE model into ECPL model, (**b**) global mesoscopic ECPL model structure of real composite laminate.

Macroscopic models are commonly employed to investigate the mechanical behavior of plain woven composites subjected to LVI loading conditions. However, if the macroscopic model is built by directly extending the mesoscopic model, it will consume a lot of work and time for calculation, which limits the application of multiscale methods in the field of composites. For precise and efficient prognostication of the mechanical properties of EWL, a reasonably accurate ECPL model was developed by rationally simplifying the local mesoscopic RVE woven structure [33], as shown in Figure 5a.

The ECPL model construction process consisted of three steps. Firstly, the yarns oriented along the warp and weft in the mesoscopic RVE, as well as the adjacent matrix, were segregated into two monolayers—aligned at 0° and 90°—corresponding to their fiber orientations, as shown in Figure 5a. Then, six loading methods were applied to the mesoscopic RVE through periodic boundary conditions for calculation, and Python scripts were deployed to derive the effective stress–strain profiles for the monolayers oriented to 0° and 90°. Finally, the mechanical property parameters of the 0° and 90° monolayers were obtained, as shown in Table 4.

In this study, a 10 mm EMAA stitch spacing was used to verify the validity of the multiscale model, as shown in Figure 5b. Based on the stitch spacing and structural features, the region in the figure was extracted to construct the global mesoscopic model. The global mesoscopic model consisted of the local mesoscopic RVE and the unaffected region of the stitch. The global mesoscopic RVE model was constructed by adding the unstitched plain woven composite structure around the local mesoscopic RVE; it also was transformed by ECPL. This methodology enables more accurate simulation and prediction of the composite’s global mechanical behavior by considering both the stitch-affected and unaffected regions at the mesoscopic scale. Furthermore, the global mesoscopic model could be more swiftly assembled by incorporating unstitched plain woven composites around the local mesoscopic RVE, allowing for different stitch spacing.

### 3.4. Macroscale Modeling

**Figure 6:**
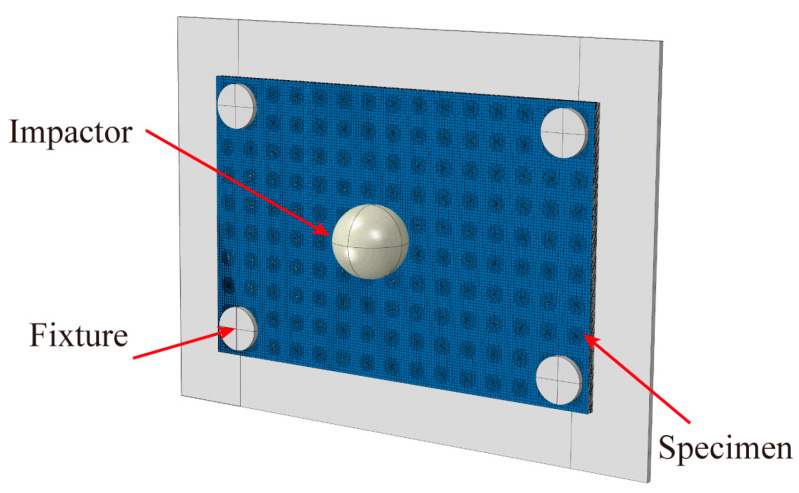
Macroscopic model used for the LVI simulation of EWL.

To holistically analyze LVI behavior and damage mechanisms in EWL, a macroscopic model was developed within the Abaqus framework based on the global mesoscopic ECPL model, as shown in Figure 6. The EWL macroscopic model consisted of 12 layers, wherein each layer was constituted by periodically arraying the global mesoscopic ECPL model in-plane. Cohesive mesh partitioning between each layer and interlayer was consistently maintained within the EWL macroscopic framework, guaranteeing nodal continuity for effective load transmission and interlayer damage capture at interface regions. The impactor, modeled as a rigid body with an 8 mm radius, was initially positioned at a 0 mm distance from the composite laminate and possessed a mass of 2.5 kg. It was imparted a specific initial velocity to achieve the targeted impact energy. The simulation incorporated dynamic analysis steps, with boundary conditions formulated to mirror the actual experimental setup.

In the macroscale model, intra-layer damage adhered to the same damage criteria established in the mesoscopic RVE model. Interlayer damage was evaluated using zero-thickness cohesive elements, with associated parameters detailed in a previous study [30].

After the first LVI test, the EWL would experience damage inside the laminate. The damaged EWL would be put into the thermoforming machine to be heated and pressured to self-healing, and the simulation of this self-healing process would be realized by the predefined field function in Abaqus. It was necessary to make appropriate settings for the impact results before self-healing. Stress, strain, and damage details for each element were stored in their corresponding ODB files, and these data given in the form of predefined fields to the repaired model as its initial state. The damaged elements were analyzed and adjusted; then, the damaged elements were replaced with EMAA to simulate the process of filling EMAA to heal the damaged area of the laminate. Through the above steps, the computational results for the first impact could be linked to the self-healing model to realize self-healing simulation after EWL impact.

## 4. Results and Discussion

### 4.1. Effect of Stitching on the Mechanical Properties of the Mesoscale RVE Model

**Figure 7:**
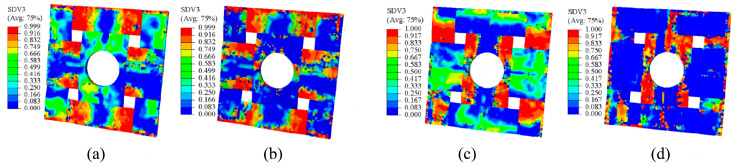
Damage according to local high-fidelity mesoscopic models under different conditions: (**a**) X-direction tension, (**b**) X-direction compression, (**c**) Y-direction tension and (**d**) Y-direction compression.

The effect of EMAA on the elastic modulus and strength properties of the composites was evaluated through finite element analysis. Within a local mesoscopic RVE, the material parameters specific to the EMAA stitching constituent were substituted with those corresponding to ethylene–acrylic acid (EAA). This computational approach was subjected to identical periodic boundary and loading conditions to derive the elastic modulus and strength properties at the mesoscopic scale. Figure 7a–d shows damage distribution in the local mesoscopic RVE model under tensile and compressive loading along the X and Y axes. The map’s numerical column values denote damage severity, achieving a state of complete damage at a value of 1. In the X-axis loading scenarios, warp fibers predominantly bear the mechanical stress. Conversely, under Y-axis tensile and compressive loading, weft fibers sustain the bulk of the stress. Warp and weft fibers are most damaged in the sinusoidal region, which is the interwoven region.

**Figure 8:**
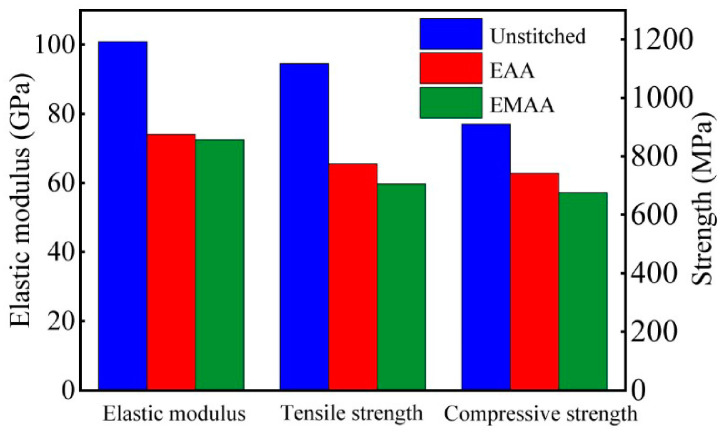
The mechanical properties according to mesoscopic RVE models of unstitched and different stitched composites.

The mechanical property parameters of EMAA and EAA are shown in Table 5, while the calculated local mesoscopic mechanical property parameters are shown in Figure 8. The unstitched mesoscopic mechanical property parameters were obtained from the simulation study conducted by Hou [34]. As can be seen in Figure 8, when thermoplastic materials with lower mechanical properties than carbon fibers were incorporated, this caused a decline in mechanical properties in the mesoscopic model. This is attributed to the fact that stitching, as a method for enhancing through-thickness reinforcement, poses a challenge in that it alters the composite structure, inadvertently reducing the in-plane mechanical properties of the composite. The addition of stitches resulted in reduced mechanical property parameters in the mesoscopic model to within 30%, which is consistent with the research findings described by Pingkarawat [35]. The mesoscopic modeling mechanical property parameters with the addition of EAA stitches are higher than those of EMAA stitches due to the higher strength of EAA itself compared to EMAA. However, EMAA in the molten state has better fluidity than EAA and flows better into damaged areas of the composite during the self-healing process.

### 4.2. Multiscale Model Validation at Different LVI Energies

EWL were subject to both experimental and numerical LVI evaluations to validate the reliability of the multiscale framework modeling. During the test, the impactor underwent free-fall motions from varying heights, resulting in three distinct instantaneous velocities: 2.83 m/s, 3.46 m/s, and 4 m/s. These velocities correspond to impact energies of 10 J, 15 J, and 20 J, respectively.

**Figure 9:**
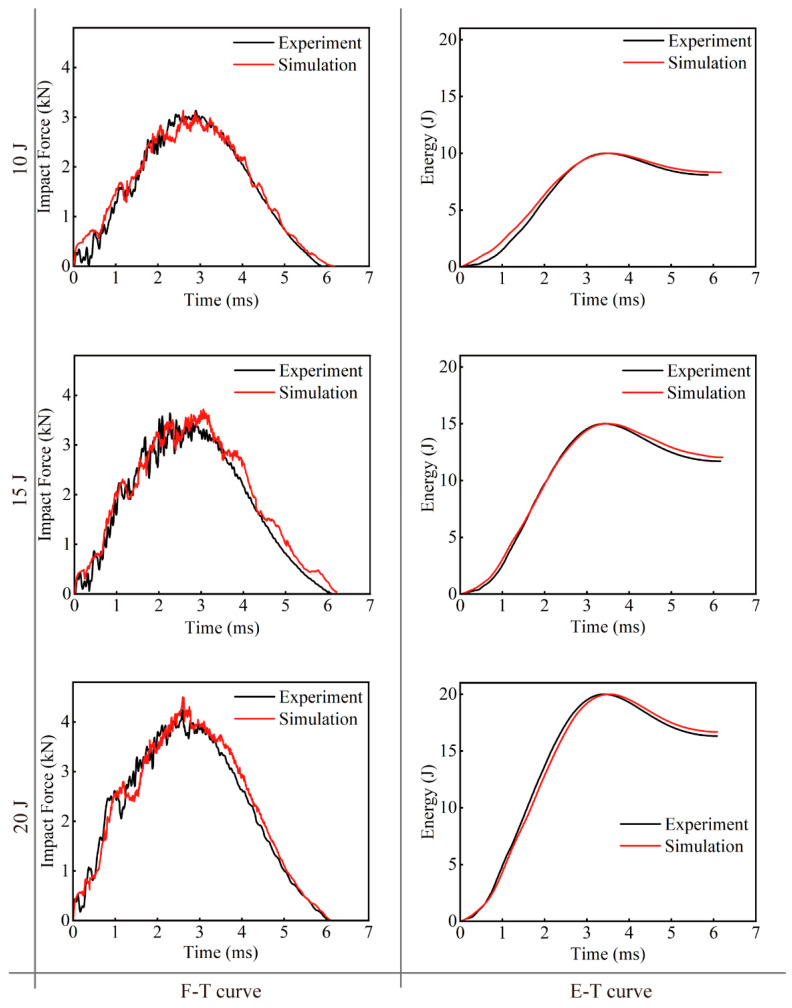
Comparison of *F*-*T* and *E*-*T* curves between numerical and experimental results of LVI tests.

According to the *F*-*T* curves in Figure 9, it can be observed that when the EWL are just in contact with the impactor, the curve rises linearly and elastic deformation occurs; damage inside the specimens has not yet appeared. With increasing impact force, the curve oscillates and rises in an oscillating manner to the peak point of the force. Cracking of the matrix, fiber breakage, delamination, etc., begin to occur in the specimens impacted. The damage within the specimens continues to expand, causing internal stresses to oscillate and the impact force to gradually reach its maximum value. As the impact force attains its peak, the downward trajectory of the impactor ends, depleting all its kinetic energy. Given that the impactor does not fully penetrate the specimens, a rebound ensues due to the reaction force exerted by the impactor, causing a subsequent decline in impact force until the end of the impact process.

Results from various impact energy tests, shown in Figure 9, indicate that as the impact energy increases, both the peak force experienced and the energy absorbed by the EWL gradually increase. Simultaneously, the oscillation amplitude of the forces sustained by the laminates also increases. The degree of damage to the EWL becomes more serious as the impact energy increases. The trends of the *F*-*T* and *E*-*T* curves in both the simulation and the experiment are substantially congruent. For the *F*-*T* curves, the observed differences are 2.59%, 2.99%, and 7.39%, respectively, all within a maximum difference of 10%. This maximum difference occurs in the 20 J impact energy case, with the experimental peak force being 4.18 kN compared to the simulated peak force of 4.49 kN. This proves the consistency between the multi-scale simulation and experimental results, thereby validating the efficacy of the multiscale strategy.

**Figure 10:**
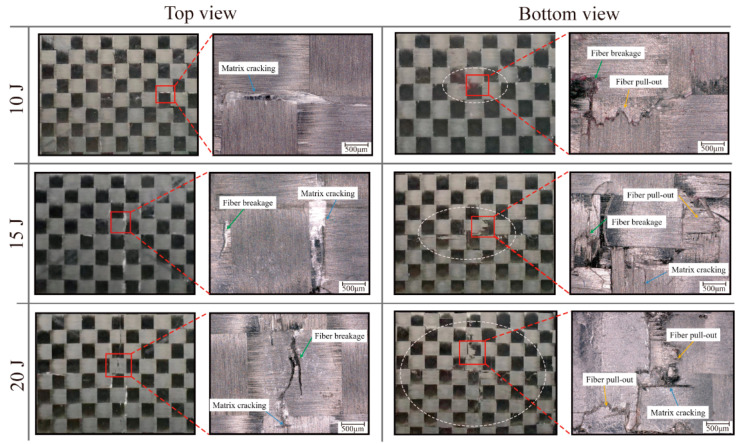
Damage morphologies of EWL from top and bottom view.

The impact damage morphology of the EWL was analyzed using a Keyence^®^ VHX 6000 (Keyence Co., Ltd., Shanghai, China) optical microscope, as presented in Figure 10. Examination of the microscopic images reveals the emergence of an elliptical damage region on both the top and bottom surfaces of the specimen, with the elliptical region becoming more elongated as the impact energy increases. The top surface manifests a diminutive indentation, whereas a subtle protrusion is evident on the bottom surface. As the impact energy increases, the degree of protrusion on the specimen’s bottom surface likewise intensifies. In the 10 J impact energy case, minor matrix cracking is observed on the top surface, while slight matrix cracking and fiber pullout occur on the bottom surface. In the 15 J impact energy case, matrix cracking and fiber breakage along the fiber direction are observed on the top surface of the specimen, while obvious fiber breakage and fiber pullout are observed on the bottom surface. In the 20 J impact energy case, the damaged area on both the top and bottom surfaces of the specimen expands. Long fiber breakage occurs on the top surface, significant fiber pullout on the bottom surface. Based on the above test results, it can be concluded that under low-energy impacts, the toughness due to the addition of EMAA stitching can absorb part of the impact energy, so that the damage on the surface of the specimen is less serious, and there is no obvious sign of damage expansion along the matrix crack. In contrast, exposure to high energy impacts induces obvious orthogonal damage along the 0° and 90° axes, indicative of complex failure modes.

**Figure 11:**
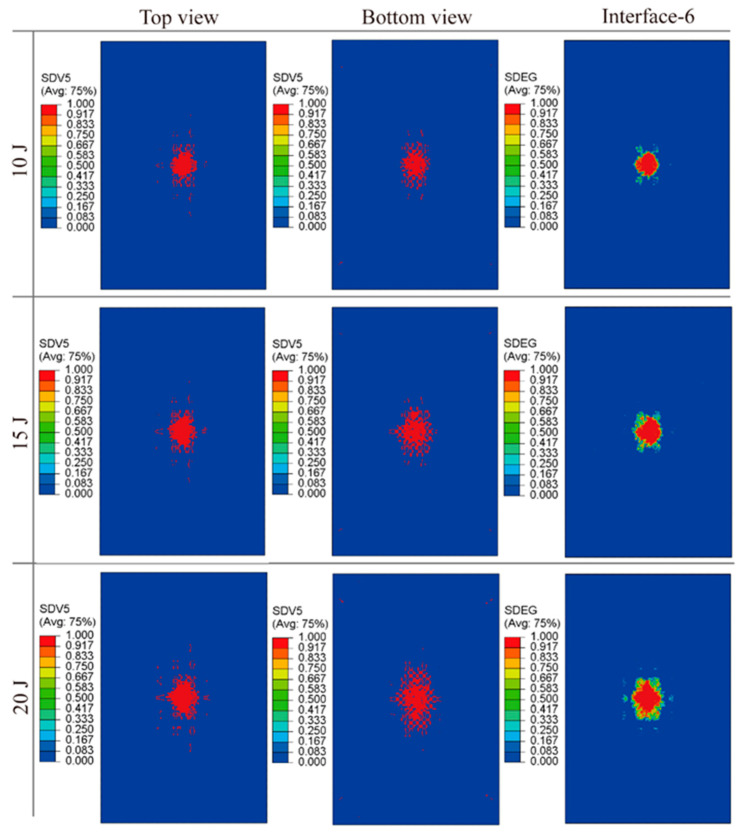
Simulated damage morphologies of EWL, top and bottom view.

The simulated damage profiles for specimens subjected to impact energies of 10 J, 15 J, and 20 J are shown in Figure 11. These reveal that the damage morphology on both the top and bottom laminate surfaces, as well as within the interlayer cohesive region, is predominantly elliptical and tends to propagate along the fiber orientation. Notably, the extent of damage is more significant on the specimen bottom surface compared to that on the top. A trend of escalating damage severity—manifesting as matrix cracking, fiber breakage, and inter-laminar delamination—is evident with increased impact energy. The similarities in location and extent between simulated and experimental impact damage validate the effectiveness of the multiscale modeling strategy.

### 4.3. LVI Mechanical Response of EWL with Different Stitch Spacings

The mechanical impact response of three types of EWL with stitch spacings of 10 mm, 15 mm and 20 mm was compared with non-EMAA stitched plain woven composite laminates (NWL). These specimens were subjected to impact tests under 15 J energy. Depending on the stitch spacing, plain woven composite structures with different area sizes unaffected by the EMAA stitches were combined around a local mesoscopic model to build a global mesoscopic model of EWL with spacing of 10 mm, 15 mm and 20 mm. Three macroscopic models were obtained from arrays of three different global mesoscopic models.

**Figure 12:**
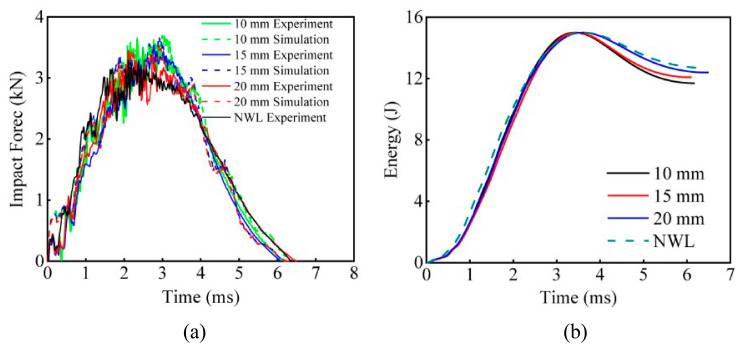
(**a**) *F*-*T* and (**b**) *E*-*T* curves of EWL with 10 mm, 15 mm, and 20 mm stitch spacings in the 15 J impact energy case.

The F-T curves of EWL for different stitch spacings are given in Figure 12. The curve trend and peak values of the simulated and experimental curves for different stitch spacings are in good coincidence. The experimental peak forces for NWL, 20 mm EWL, 15 mm EWL, and 10 mm EWL were 3.29 kN, 3.45 kN, 3.54 kN, and 3.61 kN, respectively. This indicates that the addition of EMAA stitches resulted in higher impact-resistance in plain woven composite laminates. With stitch spacings of 10 mm, 15 mm, and 20 mm, the EWL demonstrate enhancements of 9.76%, 7.55%, and 4.73%, respectively, compared to the NWL. This improvement can be attributed to the combination of EMAA stitches and carbon fiber yarn, which effectively suppressed out-of-plane deformation and limited delamination propagation. The peak impact force of EWL gradually increased as the stitch spacing became smaller and more stitches were added to limit deformation and delamination. As shown in Figure 12b, the energy absorbed by the NWL is higher than that of the EWL at each stitch spacing, and all absorbed energy is converted into damage to the plain woven composite laminate. Moreover, the smaller the stitch spacing, the less energy absorbed and the lower the extent of the damage. These cases show that the impact-resistance of EWL increases with decreasing stitch spacing within a certain range of stitch spacing.

**Figure 13:**
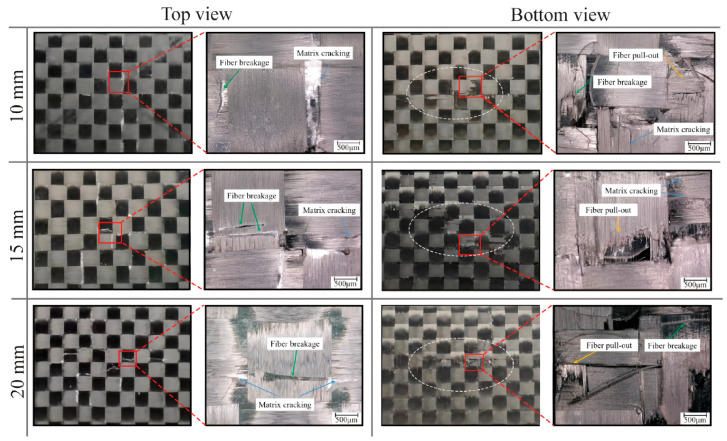
The damage morphology of EWL with 10 mm, 15 mm, and 20 mm stitch spacings in the 15 J impact energy case.

The damage morphology for the three stitch spacings of EWL in the 15 J impact energy case is shown in Figure 13. The impacts on EWL at all three stitch spacings exhibit obvious matrix cracking (white crack lines) on the top surface, along with a minor level of fiber breakage. On the bottom surface, there are instances of fiber breakage and fiber pullout. Notably, there is a remarkable resemblance in the damage pattern for these three stitch spacing cases. However, as the stitch spacing increases, the matrix cracking on the top surface of the EWL becomes more serious. This demonstrates that a smaller EMAA stitch spacing can effectively suppress damage extension.

### 4.4. Mechanical Impact Response of EWL with Different Stitch Spacings after Self-Healing

After the end of an impact, the impacted EWL specimen was placed in the thermoforming machine and heated to 150 °C for 30 min to ensure that EMAA was completely molten and could flow into the damaged region. Subsequently, the self-healing process was completed by low-pressure consolidation at room temperature for 10 min under 20 kPa. The healed EWL specimen was subjected to a secondary impact experiment under 15 J energy. At the same time, another impacted EWL specimen with 10 mm stitch spacing that had not undergone self-healing was also subjected to the secondary impact experiment to comparatively evaluate the self-healing efficacy of the EWL. In Abaqus, material substitution in the damaged area was accomplished through the use of predefined fields. Subsequently, a follow-up simulation was carried out for the 15 J impact energy case.

**Figure 14:**
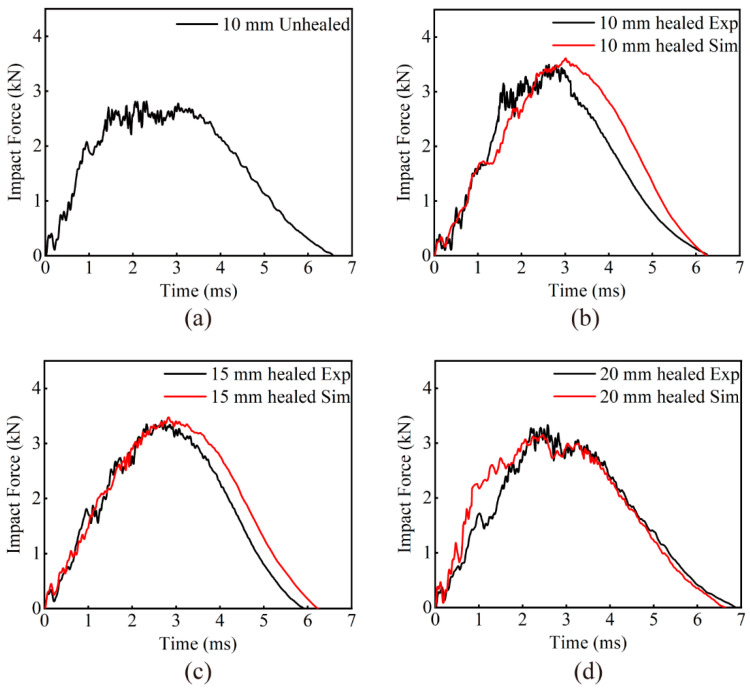
Secondary impact *F*-*T* curves of EWL with different stitch spacings at 15 J energy: (**a**) 10 mm EWL unhealed experimental curve, and experimental and simulation curves of EWL after self-healing: (**b**) 10 mm, (**c**) 15 mm, (**d**) 20 mm.

Comparing the impact *F*-*T* curves in Figure 14a,b, the curves for the secondary impact on the unhealed EWL with 10 mm stitch spacing differ significantly from the typical impact *F*-*T* curves for composites. The impact force increases rapidly after the beginning of the impact. For the following 0.003 s, the impact force oscillates up and down in a range, without forming a significant impact force peak; the maximum force is significantly lower than that of the first impact peak. This is because the laminate had already suffered significant damage during the first impact, with matrix cracking, fiber breakage, and interfacial peeling. This damage resulted in a significant reduction in the strength and stiffness of the plain woven composite laminate, leading to failure to withstand the secondary impact load and form the typical significant impact force peaks.

As illustrated in Figure 14b–d, the *F*-*T* curves derived from both experimental observations and simulation models for the self-healed EWL subjected to an impact energy of 15 J exhibit congruent trends. Remarkably, the peak discrepancies between the two sets of data fall within a tight 8% margin. It is noteworthy that under all three distinct stitch spacing configurations, the simulated response curves manifested less oscillatory behavior compared to their experimental analogs. This discrepancy may be attributed to the idealized assumptions implemented in the simulation, specifically regarding the frictional interactions between the guide rail and the impact apparatus, as well as the presumption of complete restoration of the damaged area during the self-healing process.

When subjected to an impact energy of 15 J, the peak force during the secondary impact on the unhealed EWL decreased by 20% relative to the peak force of the initial impact, as listed in Table 6. This suggests severe internal delamination damage even before the subsequent impact, leading to a substantial decrease in impact-resistance. In contrast, after undergoing self-healing at any stitch spacing, EWL exhibited only a minor reduction in peak force under the same 15 J impact energy compared to their first impact. Remarkably, they retained over 94% of their impact-resistance, implying effective healing of the initial damage. Additionally, a smaller stitch spacing was found to enhance the recovery of impact-resistance, indicating that within a specific stitch spacing range, higher EMAA content improves EWL self-healing efficiency.

**Figure 15:**
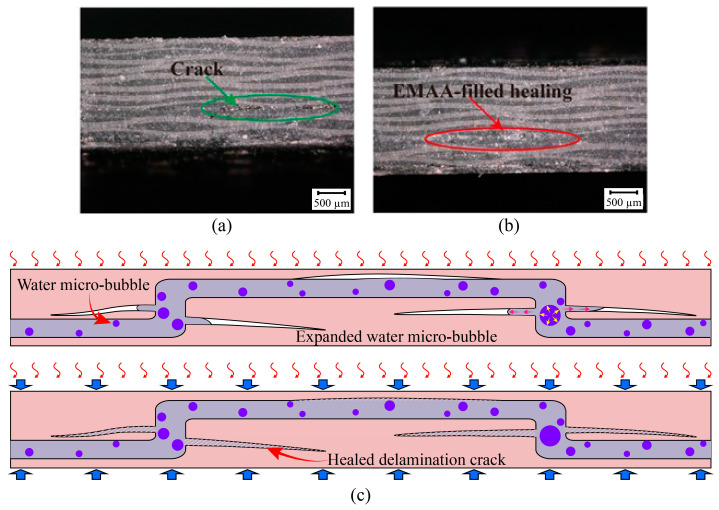
Cross-section of specimens before and after self-healing: (**a**) crack caused by impact, (**b**) EMAA flow to the area of damage, (**c**) schematic illustration of the healing mechanism.

Figure 15a shows the cross-section of an EWL specimen after the first impact. There are obvious cracks caused by the first impact inside the EWL specimen, as shown in Figure 15a. Figure 15b showcases a cross-sectional perspective of another EWL specimen after undergoing the self-healing process. In this case, thermally activated and pressurized EMAA flows through persistent three-dimensional self-healing channels, effectively healing the localized damage within the EWL specimen. Figure 15c reveals the healing mechanism of EMAA. When subjected to heat treatment, EMAA filaments reach a molten state, prompting a condensation reaction between the acid groups within the polymer and the amine groups present in the epoxy resin. This reaction leads to the release of water molecules and the formation of microbubbles within the EMAA. These microbubbles exert internal pressure within molten EMAA under high-temperature conditions. Consequently, molten EMAA expands outward under the high pressure driven by the internal pressure generated by these microbubbles, thereby establishing a pressure-driven delivery mechanism. This process facilitates the delivery of EMAA into the damaged delamination crack. As shown in Figure 14b–d, the impact F-T response curve of the EWL after self-healing still maintains a typical impact response, indicating that the damage formed after the first impact can be filled and healed effectively by melted EMAA.

## 5. Conclusions

This study employs a novel multiscale modeling approach based on local homogenization to predict impact response and damage behavior in EWL. Numerical simulation models were developed at three scales: microscopic, mesoscopic and macroscopic. At the mesoscopic scale, the mesoscopic model was constructed by extracting the EMAA stitch filament and its surrounding composite laminate structure, and computational efficiency was improved by the ECPL method while ensuring prediction accuracy. The mesoscopic model was arranged periodically to build the macroscopic LVI model. The impact responses of EWL before and after self-healing were investigated to explore their damage behavior and healing mechanisms. Conclusions are summarized below:

- *F*-*T* curves obtained from LVI experiments were compared with simulation calculations. The analysis reveals a high degree of congruence between the two sets of data, with discrepancies confined within an 8% margin. This substantiates the efficacy of the multiscale approach applied to the EWL model and indicates that matrix damage and delamination constitute the primary failure mechanisms.
- In the 15 J impact energy case, EWL with different stitch spacings offer enhanced performance in absorption of peak impact force and energy compared to NWL. The peak impact force increases to 0.31 kN with decreasing stitch spacing. The smaller the stitch spacing, the better the impact-resistance of the composite laminates.
- After impact damage, EWL underwent a thermal compression process that activated the internal three-dimensional self-healing network. In the molten state, EMAA flowed into the damaged area and healed the cracks. The self-healing efficiency of EWL increased with decreasing stitch spacing, up to 98.28%. This effectively demonstrated the ability of EMAA to heal composite laminate damage.

Overall, the multiscale modeling approach performed enables the robust prediction of impact-resistance and self-healing efficiency in EWL composites. Moreover, the modeling approach shows promising engineering applications given its high accuracy and efficiency. In future work, this approach could be extended to numerically reveal the damage mechanisms in various high-performance self-healing composites.

## Figures and Tables

**Table 1:** Mechanical parameters of carbon fiber and epoxy resin. Superscripts *f* and *m* represent fiber and matrix, respectively.

Carbon Fiber T300/3K		Epoxy Resin 7901	
$\mathrm{Young}’s\;\mathrm{modulus},\;E_{11}^{f}$/GPa	230	$\mathrm{Young}’s\;\mathrm{modulus},\;E^{m}$/GPa	3.5
$\mathrm{Young}’s\;\mathrm{modulus},\;E_{22}^{f}$$,\;E_{33}^{f}$/GPa	40	$\mathrm{Shear}\;\mathrm{modulus},\;G^{m}$/GPa	1.3
$\mathrm{Shear}\;\mathrm{modulus},\;G_{12}^{f}$$,\;G_{13}^{f}$/GPa	24	$\mathrm{Poisson}’s\;\mathrm{ratio},\;\upsilon^{m}$	0.35
$\mathrm{Shear}\;\mathrm{modulus},\;G_{23}^{f}$/GPa	14.3	$\mathrm{Tensile}\;\mathrm{strength},\;M_{T}^{m}$/MPa	112
$\mathrm{Poisson}’s\;\mathrm{ratio},\;\upsilon_{12}^{f}$ $,\;\upsilon_{13}^{f}$	0.26	$\mathrm{Compressive}\;\mathrm{strength},\;M_{C}^{m}$/MPa	241
$\mathrm{Poisson}’s\;\mathrm{ratio},\;\upsilon_{23}^{f}$	0.44	$\mathrm{Shear}\;\mathrm{strength},\;M_{s}^{m}$/MPa	89.6
$\mathrm{Tensile}\;\mathrm{strength},\;F_{T}^{f}$/MPa	3258	$\mathrm{Fracture}\;\mathrm{energy},\;G_{IC}^{m}$/(N/mm^2^)	1
$\mathrm{Compressive}\;\mathrm{strength},\;F_{C}^{f}$/MPa	2470		
$\mathrm{Fracture}\;\mathrm{energy},\;G_{IC}^{f}$/(N/mm^2^)	12.5		

**Table 2:** Equivalent properties of the microscale RVE model. Superscript *y* represents fiber-bundle yarn.

Elastic Properties		Damage Properties	
$\mathrm{Young}’s\;\mathrm{modulus},\;E_{11}^{y}$/GPa	184.77	$\mathrm{Tensile}\;\mathrm{strength},\;X_{T}^{y}$/MPa	2890
$\mathrm{Young}’s\;\mathrm{modulus},\;E_{22}^{y}$$,\;E_{33}^{y}$/GPa	19.17	$\mathrm{Compressive}\;\mathrm{strength},\;X_{C}^{y}$/MPa	2059
$\mathrm{Shear}\;\mathrm{modulus},\;G_{12}^{y}$$,\;G_{13}^{y}$/GPa	8.46	$\mathrm{Tensile}\;\mathrm{strength},\;Y_{T}^{y}$/MPa	92
$\mathrm{Shear}\;\mathrm{modulus},\;G_{23}^{y}$/GPa	14.3	$\mathrm{Compressive}\;\mathrm{strength},\;Y_{C}^{y}$/MPa	185
$\mathrm{Poisson}’s\;\mathrm{ratio},\;\upsilon_{12}^{y}$ $,\;\upsilon_{13}^{y}$	0.27	$\mathrm{Shear}\;\mathrm{strength},\;s_{12}^{y}$$,\;s_{13}^{y}$/MPa	107
$\mathrm{Poisson}’s\;\mathrm{ratio},\;\upsilon_{23}^{y}$	0.42	$\mathrm{Shear}\;\mathrm{strength},\;s_{23}^{y}$/MPa	59

**Table 3:** Geometric parameters of local mesoscopic RVE model.

Parameter	*l*	*h*	*D*	*b*	*a*	*w*	*t*
Value/mm	4	0.25	1.2	1.2	0.4	1.62	0.11

**Table 4:** Equivalent mechanical parameters for 0°and 90° monolayers in ECPL modeling.

Parameter	0° Monolayer	90° Monolayer
Young’s modulus, *E*_11_/GPa	70,075	67,840
Young’s modulus, *E*_22_, *E*_33_/GPa	13,299	13,405
Shear modulus, *G*_12_, *G*_13_/GPa	1440	1632
Shear modulus, *G*_23_/GPa	2325	2148
Poisson’s ratio, *υ*_12_, *υ*_13_	0.13	0.13
Poisson’s ratio, *υ*_23_	0.43	0.4
Tensile strength, *X_T_*/MPa	705	658
Compressive strength, *X_C_*/MPa	675	587
Tensile strength, *Y_T_*/MPa	182	172
Compressive strength, *Y_C_*/MPa	76.2	92.4
Shear strength, *S*_12_, *S*_13_/MPa	30	36
Shear strength, *S*_23_/MPa	87	83

**Table 5:** Mechanical property parameters of EAA and EMAA.

Parameter	EAA	EMAA
Tensile modulus, *E*/MPa	33.1	22.3
Tensile strength, *X*/MPa	18.6	16
Poisson’s ratio, *υ*	0.4	0.42
Density, *ρ*/(g/cm^3^)	0.91	0.90

**Table 6:** The impact peak force and healing efficiency after healing.

Energy/J	Peak Force/N		Healing Efficiency
15	Unhealed	2814	/
	10 mm	3551	98.28%
	15 mm	3447	97.26%
	20 mm	3268	94.67%

## Data Availability

The original contributions presented in the study are included in the article, further inquiries can be directed to the corresponding author.
